# Editorial: New Methods for Red Blood Cell Research and Diagnosis

**DOI:** 10.3389/fphys.2021.755664

**Published:** 2021-09-29

**Authors:** Paola Bianchi, Richard van Wijk

**Affiliations:** ^1^Hematology Unit, Pathophysiology of Anemias Unit, Foundation IRCCS Ca' Granda Ospedale Maggiore of Milan, Milan, Italy; ^2^Central Diagnostic Laboratory-Research, University Medical Center Utrecht, Utrecht University, Utrecht, Netherlands

**Keywords:** red blood cell, anemia, diagnostics, ektacytometry, next generation sequencing, metabolomics (OMICS), sickle cell disease, RBC membrane defects

The last few years have been a challenging and exciting period for the study of RBC pathophysiology and related defects associated with hemolytic anemia. In fact, these disorders, regarded as hematological niche disorders, have today aroused greater interest thanks to the availability of new technological and therapeutical approaches, such as gene therapy for sickle cell disease (SCD) (Orkin and Bauer, 2019) and thalassemia (Boulad et al., 2018) or more recently small molecule activator therapy for pyruvate kinase deficiency (Grace et al., 2019). In this context, the need of a more precise diagnosis becomes crucial, accelerating the improvement of diagnostic procedures and differential diagnosis by developing new methodologies to investigate RBC properties in normal and pathological conditions.

This Research Topic has been specifically designed to give rooms to new experimental and diagnostic approaches for the study of red blood cells. The success of this Research Topic, with 15 articles accepted for publications and ranging from topics that address basic science, diagnosis and therapy as well confirms the growing interest of the scientific community on these themes.

In an extensive review by Russo et al., advantages and disadvantages of Next Generation Sequencing (NGS) are critically reviewed, focusing on diagnosis and basic research of rare hemolytic anemias and considering the future perspectives in this era of precision medicine. There are several approaches to this molecular testing, including custom-designed targeting panels (t-NGS), whole-exome sequencing (WES), or wide genome sequencing (WGS), the choice of which depends on phenotyping, genetic heterogeneity, and gene size. For patients who show complete phenotyping, single-gene testing remains recommended. The use of NGS, also allows the identification of new causative genes, and of polygenic conditions and genetic factors that modify disease severity of hereditary anemias. As a consequence, NGS has been adopted by many expert centers for hemolytic anemias as part of the diagnostic work-up. In a monocentric study, the 3 years' experience with targeted-NGS platform (consisting of 43 genes) used in the diagnosis of congenital hemolytic anemias has been critically evaluated by Fermo et al. One hundred and twenty two patients were investigated, and the results compared with that of a conventional laboratory diagnostic workup. The method was able to establish the diagnosis in 74% of patients with a diagnostic workup based on laboratory testing, and in an additional 35% of patients that were undiagnosed after extensive hematologic investigations. This indicates that rare and ultra-rare RBC diseases definitely benefits of t-NGS approaches. This is for example the case for a new variant of *ALAS2* gene associated with sideroblastic anemia described by Lira Zidanes et al. Iron loading anemias are characterized by ineffective erythropoiesis and iron overload and in the presented case iron parameters were discussed in light of the atypical clinical presentation and genotype. Finally, a combined t-NGS and WES approach has been used to characterize a series of five patients with unstable hemoglobinopathies, as reported by Rizzuto et al. Also this group of rare disorders represents a diagnostic challenge due to its rarity, the dominant pattern of inheritance and the occurrence of *de novo* variants. The inclusion of α and β-globin genes in routine NGS approaches for rare anemia disorders has to be considered to improve the diagnostic efficiency of rare anemias.

The recent developments in high-throughput omics approaches paved the way to perform proteomic and metabolomic analys of RBCs, focusing on differences between normal and pathological conditions. RBCs offer in fact an interesting model to study cellular metabolism. Moreover metabolomics may be used to better understand the changes that occur during storage of blood for transfusion purposes. Zoomics is a new branch of metabolomics that is uses to investigate the blood metabolome across species. Bertelone et al. provide the here first comparative metabolomics analysis of fresh and stored human, baboon, and macaque RBCs. The results indicated similarities and differences across species, which ultimately resulted in a differential propensity to undergo morphological alterations and lysis as a function of the duration of refrigerated storage.

The pathophysiological basis of RBC membrane channel defects has only recently been described, establishing the molecular basis that underlies hereditary dehydrated (*PIEZO1*) and overhydrated (*RhAg*) forms of hereditary stomatocytosis, and some other rarer forms (*KCNN4, GLUT1, ABCB6*, and *ABCB5*). Most of the causative mutations encode gain of function variants and a very tight interplay among specific and non-selective channels has been described. Recent studies also confirm that hereditary stomatocytosis is often associated with a certain degree of dyserythropoiesis. *PIEZO1* expression for example is not limited to RBC but its expression levels are significantly higher in erythroid precursors. In a study by Aglioro et al. it was observed that integrin α4β1 and α5β1 present on erythroblasts facilitate *PIEZO1* interactions in erythroblastic islands. Chemical activation of *PIEZO1* leads to increased adhesion to VCAM1 and fibronectin in flowing conditions, suggesting an inside-out activation of integrin on erythroblasts. This phenomenon seems to be facilitated by calcium-dependent activation of Ca^2+^-dependent protein kinase C and Calpain. This study suggests a novel involvement of Ca^2+^ signaling during erythropoiesis. From a diagnostic point of view, Gardos channelopathies are often difficult to be diagnosed due to the absence of specific laboratory markers of the disease. A novel and quick approach to differentiate between *KCNN4* and *PIEZO1* variants, allowing to rapidly target these patients for gene analysis, has been proposed by Picard et al. For this they used automated reticulocytes parameters obtained from an ADVIA 2120 (Siemens®) analyzer, in particular the reticulocyte mean corpuscular volume (rMCV) and mean corpuscular hemoglobin concentration (rMCHC). rMCV was found to be significatively smaller than MCV and rMCHC higher than MCHC in 15 *KCNN4* mutated patients vs. 79 *PIEZO1* cases. Cut-off values were proposed to obtain a 100% sensitivity and specificity, regardless of age, mutation or splenectomy status.

Ektacytometry is a technique that is more and more commonly applied in diagnostic approaches for red blood cell disorders. In particular osmotic gradient ektacytometry is now considered the gold standard for the diagnosis of red blood cell membrane disorders, such as hereditary spherocytosis (HS), and hydration disorders such as *PIEZO1*-defective hereditary xerocytosis. Very recently a new form of ektacytometry, oxygen gradient ektacytometry, was developed that especially holds promise for patients with SCD. This technique measures red blood cell deformability under deoxygenation and reoxygenation, thereby capturing the dynamic process of red blood cell sickling. In this issue, Sadaf et al. evaluate the correlation between oxygen gradient ektacytometry-derived parameters and “classical” biomarkers in the field of SCD such as levels of fetal hemoglobin and the percentage of dense RBCs. They convincingly show that individual parameters correlate with known biomarkers of SCD severity. In addition, they demonstrate that oxygen gradient ektacytometry assesses the cumulative effect of known biomarkers and, likely, additional factors that contribute to sickling. Therefore, their findings further establish this technique as a novel useful biomarker for clinical severity and treatment efficacy in the field of SCD. Berrevoets et al. explore another, relatively unknown, form of ektacytometry. This cell membrane stability test (CMST) was used in combination with osmotic gradient ektacytometry to study the longitudinal effects of splenectomy on RBC characteristics in a small cohort of HS patients. They show that before splenectomy the composition of the RBC population is more heterogeneous, with RBC that are more rigid and have increased intracellular viscosity and reduced deformability. Splenectomy improves cellular hydration status and allows cells to regain the ability to shed membrane. The latter was assessed using the CMST and the authors postulate that this yet-undescribed RBC feature reflects RBC membrane health, and as such the CMST holds promise as a novel biomarker for clinical severity and phenotypic expression in HS.

Eosin-5-maleimide binding to extracellular proteins of the RBC membrane is a very specific test for the diagnosis of HS. In an attempt to further optimize this commonly used test Glenthøj et al. circumvent the need for blood samples from healthy control individuals by using commercially available fluorescent beads in performing this test. By analyzing a large cohort of HS patients, using osmotic gradient ektacytometry as a gold standard, they found that the EMA binding test results were not compromised by this modification of the EMA test and an accuracy of 90.3% was obtained (vs. 88.6% for the “classical” version of the test). Since fluorescent beads are more stable and better standardized, this modification represents an attractive alternative of the EMA binding test, also allowing for interlaboratory comparisons and quality control programs.

Porro et al. extend the use of ektacytometry into the field of cardiovascular diseases by investigating the association between RBC morphodynamic features, i.e., aggregability and deformability, in patients with different grades of coronary stenosis. They calculated a global RBC morphodynamic score and a related risk chart, which was associated with the extent of high-risk plaque features. In a cohort of nonobstructive coronary artery disease patients positive correlations were found between RBC rigidity, osmotic fragility or aggregability and high-risk plaque features. This led them to conclude that RBC morphodynamic features may be used in the identification of high-risk patients in this specific patient category.

The disadvantage of osmotic gradient ektacytometry is that the technique assesses RBC deformability of the entire RBC population, not individual RBCs. Therefore, the development of new diagnostic tools able to analyze a statistically relevant number of single cells would provide important complementary information. Faivre et al. have addressed this issue by evaluating the mechanical response of artificially altered RBCs and RBCs from HS and SCD patients flowing through a microfluidic constriction. Comparison of the differences in extension at the exit and the shape recovery time between healthy and chemically altered RBCs provide a direct signature of the RBC membrane composition and architecture. Further analysis of HS and SCD RBCs demonstrate a first proof-of-principle that a passive microfluidic approach could be used to discriminate between the two diseases, thereby warranting further study of this technique for diagnostic and prognostic purposes.

Another example of single cell analysis is Raman spectroscopy. Jacob et al. used this technique to analyze the toxic effects of bilirubin on RBC. Regardless of the underlying cause, a common feature of hemolytic anemia is increased levels of bilirubin, the breakdown product of hemoglobin. Using minimal-invasive sample handling procedures and using a home-built micro-Raman spectroscopy system coupled with laser-tweezers the authors obtained specific fingerprints from RBC obtained from healthy volunteers and patients with jaundice, indicating biochemical alterations resulting from increased bilirubin levels. Their study paves the way for further investigations using this technique in understanding fundamental RBC behavior in different RBC diseases.

Analysis of single RBCs as well as the whole population of RBCs was done in a study by Cloos et al. They used a vast number of techniques for a comprehensive analysis of the acantocytes present in a patient with hypobetalipoproteinemia due to a pathogenic mutation in *APOB*. In particular they wondered how, and to what extent hypobetalipoproteinemia could affect RBC functionality. The major findings led them to conclude that the RBCs in this patient, who was hematologically normal, had altered cytoskeletal and membrane lipid lateral asymmetry while deformability was only mildly impaired. Their case report study is a nice example of the potential use of membrane biophysics and lipid vital imaging as new methods in the study of RBC disorders.

Currently, many new forms of therapy are being developed for the treatment of hemoglobinopathies, in particular SCD and beta-thalassemia. Among them are therapies that aim to increase the level of fetal hemoglobin. Manchinu et al. now further explore an alternative strategy that aims to increase the levels of HbA2. They report on considerably increased levels of δ-globin mRNA in a deoxyribonuclease II-α knock out mouse model in which interferon β (IFNb) is activated. At the same time IFNb activation in the fetal liver reduces β-globin mRNA levels. They translated their findings to a cohort of patients with multiple sclerosis on IFNb treatment, in whom they detected a small but significant increase in HbA2 levels. Therefore, their study represents a first proof of principle that elevating the levels of HbA2 could be explored as a novel therapeutic option for treating β-hemoglobinopathies.

The considerable technological progress reported in this Research Topic will contribute to increasing our knowledge on RBC pathophysiology, and a better understanding of RBC disorders. In turn, this may enhance the development of new diagnostic procedures and targeted therapies for these rare disorders. Because of its success, the second volume of this Research Topic has recently been launched and is now open for submission.

## Author Contributions

PB and RvW wrote the manuscript and approved the final version. Both authors contributed to the article and approved the submitted version.

## Conflict of Interest

The authors declare that the research was conducted in the absence of any commercial or financial relationships that could be construed as a potential conflict of interest. The handling editor declared a past co-authorship with the authors.

## Publisher's Note

All claims expressed in this article are solely those of the authors and do not necessarily represent those of their affiliated organizations, or those of the publisher, the editors and the reviewers. Any product that may be evaluated in this article, or claim that may be made by its manufacturer, is not guaranteed or endorsed by the publisher.
